# Anatomic reduction of the sacroiliac joint in unstable pelvic ring injuries and its correlation with functional outcome

**DOI:** 10.1007/s00068-020-01504-z

**Published:** 2020-09-30

**Authors:** Katharina Jäckle, Christopher Spering, Mark-Tilmann Seitz, Sebastian Höller, Marc-Pascal Meier, Franziska Melanie Hahn, Mehool R. Acharya, Wolfgang Lehmann

**Affiliations:** 1 Department for Trauma Surgery, Orthopaedics and Plastic Surgery, University Medical Center Göttingen, Robert-Koch Str. 40, 37075 Göttingen, Germany; 2 Department of Trauma and Orthopaedics, North Bristol NHS Trust, Southmead Hospital, Southmead Rd, Bristol, BS10 5NB UK

**Keywords:** Sacroiliac joint fractures, Sacroiliac joint disruptions, Anatomic reduction, Percutaneous screw fixation, Long-term outcome

## Abstract

**Purpose:**

Reduction and percutaneous screw fixation of sacroiliac joint disruptions and sacral fractures are surgical procedures for stabilizing the posterior pelvic ring. It is unknown, however, whether smaller irregularities or the inability to achieve an anatomic reduction of the joint and the posterior pelvic ring affects the functional outcome. Here, the long-term well-being of patients with and without anatomic reduction of the posterior pelvis after sacroiliac joint disruptions is described.

**Methods:**

Between 2011 and 2017, 155 patients with pelvic injuries underwent surgical treatment. Of these, 39 patients with sacroiliac joint disruption were examined by radiological images and computer tomography (CT) diagnostics and classified according to Tile. The functional outcome of the different surgical treatments was assessed using the short form health survey-36 (SF-36) and the Majeed pelvic score.

**Results:**

Complete data sets were available for 31 patients, including 14 Tile type C and 17 type B injuries. Of those, 26 patients received an anatomic reduction, 5 patients obtained a shift up to 10 mm (range 5–10 mm). The SF-36 survey showed that the anatomic reduction was significantly better in restoring the patient’s well being (vitality, bodily pain, general mental health and emotional well-being). Patients without this treatment reported a decrease in their general health status.

**Conclusions:**

Anatomic reduction was achieved in over 80% of patients in this study. When comparing the long-term well-being of patients with and without anatomic reduction of the posterior pelvis after sacroiliac joint disruptions, the results suggest that anatomical restoration of the joint is beneficial for the patients.

## Introduction

Pelvic ring injuries account for approximately 3% of all fractures, and therefore are relatively rare injuries [1, 2]. However, in polytraumatized patients, nearly 25% of cases involve the pelvic ring [1, 2]. In older patients, pelvic injuries frequently occur due to poor bone quality, resulting in low energy related injury mechanism [1]. Pelvic injuries can be classified according to Marvin Tile [3] or Young and Burgess [4]. The posterior pelvic ring can be injured resulting in either fracture of the ilium, pure sacroiliac joint disruptions, transiliosacral fractures or sacral fractures. If the posterior pelvic ring is involved the sacroiliac joint is often affected [5]. The sacroiliac joint of the pelvis consists of two distinct joint connections. The posterior-superior two thirds of the joint represent a syndesmosis, i.e. a bone union through connective tissue, and the anterior-inferior third is typical of a synovial joint [5]. The sacroiliac joint is difficult to visualize on plain radiographs due to its obliquity. Computed tomography (CT) can be used to help determine the extent of the fracture and clarify the extent of injury to the posterior pelvic ring. The synovial sacroiliac joint is usually about 2–4 mm wide [5] and normally exhibits a symmetrical appearance. Sacroiliac joint disruptions can represent a rupture of the ligamentous joint associated with type B or C pelvic injuries [3, 4]. However, although fundamental differences allow to distinguish between type B and C injuries [3, 4], differences concerning the outcome of their surgical treatment (*p* = 0.055) were not observed. Thus, it was referred to a sacroiliac joint disruption if the sacroiliac joint width exceeds 4 mm and lacks its normal symmetrical appearance as determined by CT imaging. According to these criteria pure sacroiliac disruptions are rare, but not yet described as such in the literature. Multiple studies have shown that reduction and stabilization of sacroiliac joint disruptions affect the outcome, however in the majority of these studies it is concluded that reduction to within 5 mm of the opposite unaffected side or the upper end of the width of the native joint (4 mm) is not associated with an adverse outcome [5, 6]. This suggests that residual malreduction, even a minimal one, is associated with an adverse clinical and functional outcome. The present study investigates the reduction of sacroiliac joint disruptions and correlates reduction with clinical and functional outcome.

## Methods

### Patient collective for study

The present study was performed in compliance with the Helsinki Declaration and approved by the ethics committee of the University Medical Center Göttingen (approval number: AN 3/8/19).

Inclusion criteria for this retrospective observational study with prospective longitudinal study share were defined by a patient collective at the University Medical Center Göttingen in the period 2011–2017 with traumatic sacroiliac joint disruptions. Out of a total of 155 patients with pelvic injuries that underwent surgery during this time, 39 patients with sacroiliac joint disruptions of the posterior pelvic ring (mean age: 47.15; 14 females and 25 males) were included in the study. The pelvic injury was assessed by CT diagnostics and X-ray images both preoperatively and postoperatively. Preoperatively the injuries were classified according the Tile classification. The post-operative images were analyzed by two experienced pelvic surgeons and the reduction was documented as being anatomical or non-anatomical. The reduction was deemed anatomical if the sacroiliac joint had a symmetrical appearance in the CT diagnosis and the sacroiliac joint did not exceed a width of 4 mm [5, 6].

### Surgical procedure

All patients in the study had traumatic sacroiliac joint disruptions, which were stabilized by percutaneous fixation (see Table 1). They were selected on the basis of operations and procedures key (OPS) coding (5–79a.0e; 5–79b.0e; 5–790.0d; 5–798.3) and international classification of diseases (ICD)-10 coding (S33.2). The injury patterns were essentially traffic accidents (see Table 2). The patients were retrospectively interviewed with a precasted questionnaire. The well-being of the patients was scored by interviews which took place in 2019, i.e. 2 years after the last CT scans were obtained. Corresponding CT images were taken preoperatively and directly postoperatively. They were taken to select the patients to be interviewed. The written voluntary disclosure of confidential information, the corresponding X-ray images and CT diagnostics, were handled and evaluated anonymously. The 39 selected patients were contacted and 31 of them (79%) responded back with completed questionnaires. Of these, 26 patients had an anatomic reduction of the sacroiliac joint (mean age: 46.62; 8 females and 18 males) and 5 patients had a non-anatomic reduction (mean age: 53.40; 3 females and 2 males) (see Table 3). Complete data was available for 31 patients, 14 Tile type C and 17 type B injuries. Although in the awareness of fundamental differences that distinguishes type B and C injuries [3, 4], no severe differences concerning the outcome of their surgical treatment (*p* = 0.055) were noted. Thus, we combined the B and C types and referred to them as pelvic injuries.

**Table 1:** Gender distribution

Number of female patients	11
Percent of female patients [%]	35.48
Number of male patients	20
Percent of male patients [%]	64.52
Ratio (males/females)	1.82

**Table 2:** Causes of accidents of the population with anatomic and non-anatomic reduction

Crash trauma (*n* = 6) [%]	19.35
Riding accident (*n* = 2) [%]	6.45
Traffic accident (*n* = 16) [%]	51.61
Spill (*n* = 1) [%]	3.23
Rollover trauma (*n* = 1) [%]	3.23
Entrapment (*n* = 1) [%]	3.23
Unknown (*n* = 4) [%]	12.90

**Table 3:** Baseline characteristics of the populations

Anatomic reduction	
Number of patients	26
Age range [years]	5–80
Age mean [years] ± SEM	46.62 ± 18.96
Gender	8 females
	18 males
Stationary stay range [days]	5–95
Stationary stay mean [days] ± SEM	32.50 ± 21.79

Non-anatomic reduction	
Number of patients	5
Age range [years]	39–75
Age mean [years] ± SEM	53.40 ± 14.05
Gender	3 females
	2 males
Stationary stay range [days]	17–43
Stationary stay mean [days] ± SEM	28.00 ± 9.49

Shortly after the trauma, all patients underwent closed reduction of the posterior pelvic ring and percutaneous sacroiliac screw osteosynthesis by inserting one or two cannulated screws into the sacrum at the level of S1 and/or S2 under radiological control (see Table 1). Cannulated screw with thread diameter between 7.3 and 7.5 mm and a length between 75 and 110 mm with a washer were used for osteosynthesis (Axomed GmbH, Freiburg, Germany). The anterior pelvic ring was stabilized as necessary.

According to the standard clinical care X-ray controls were acquired prior surgery, postoperatively on the second day and after three and six weeks before starting the rehabilitation. The X-ray controls included an antero-posterior view of the pelvis with additional inlet/outlet projections. In addition, CT scans were performed both preoperatively and postoperatively to determine the reduction of the sacroiliac joint and the osteosynthesis material.

### Questionnaire and evaluation of the questionnaire

All patients were contacted by sending a scripted questionnaire consisting of the Majeed Pelvic score and SF-36 [7, 8]. In addition, patients were asked about tenderness over the sacroiliac joint in the form of a numerical rating scale (NRS). The NRS represents a one-dimensional metric scale to score the intensity of pain. It consists of a sequence from zero (no intensity) to 10 (strongest intensity) to be subjectively judged by the patient.

### Statistics

Statistical analysis was performed using the D’Agostino-Pearson test to check for normal distribution. The significance calculation was based on the Wilcoxon-Mann–Whitney test and the significance level was set to alpha = 5%. Furthermore, the Pearson correlation test was performed and the confidence interval was set to 95%, for all statistical tests using the statistics software Graphpad Prism 8 (version 8.1.1 for mac).

## Results

**Fig. 1:**
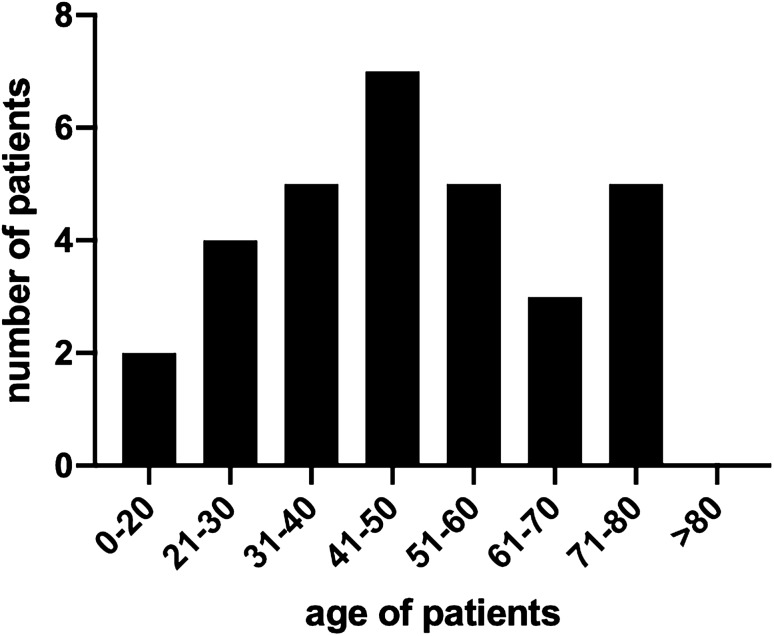
Age distribution and number of the two patient collectives in the overview. Age distribution of the patient collective with and without anatomically correct reduction. *n* = 31 [(*n* (anatomically correct) = 26; *n* (not anatomically correct) = 5]

Thirty-nine consecutive patients (mean age: 47.15 years (range 39–75); 14 females and 25 males) with sacroiliac joint disruptions were included in this retrospective observational study of prospectively collected data. Thirty-one of thirty-nine patients (79%) returned the fully completed questionnaire. Figure 1 shows the age distribution and number of patients examined in this study.

**Fig. 2:**
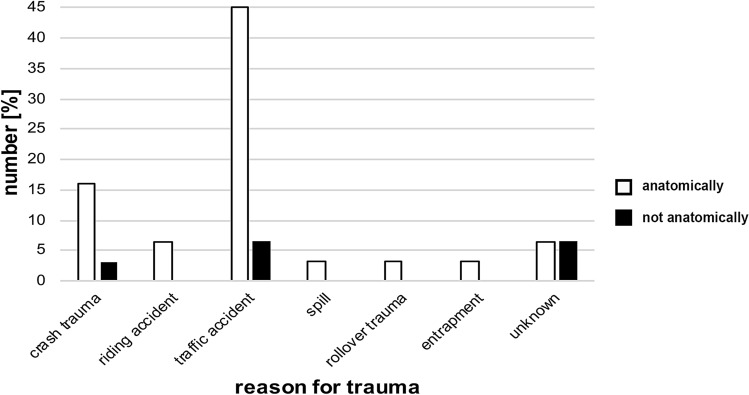
Reason for trauma of the two patient collectives in the overview. Reason for trauma of the patient collective with numbers in % for anatomically correct reduction (white bars) and not anatomically correct reduction (black bars)

Table 2 and Fig. 2 show the mechanism of injury. The most common cause of injury was traffic accidents involving cars, trucks, bicycles and motorbikes also including pedestrians. The second most common cause were injuries associated with a fall from a height of more than 2.5 m (“crash trauma”, e.g. fall from a balcony, paragliding, scaffoldings of buildings, ladder) and riding accidents. The remaining injuries were caused by spills (refers to an accident that for example results in a burying with slipping off rocks), entrapment/crushing trauma (refers to crushing/squeezing of the pelvis by heavy objects) and a trauma caused for example by a rollover accident (Table 2; Fig. 2).

**Fig. 3:**
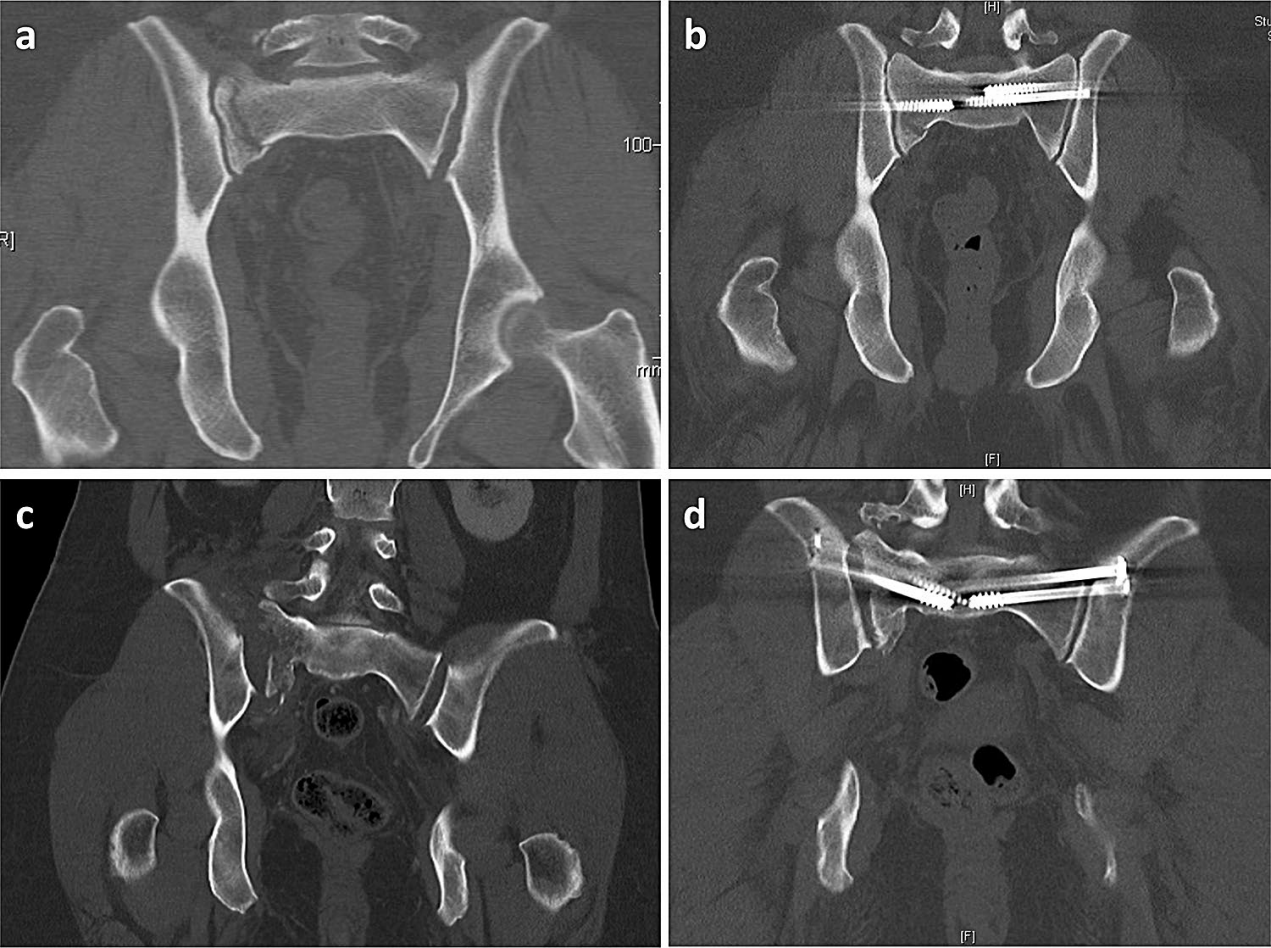
CT picture of two patients showing bilateral pelvic injuries before and after the surgery. **a** CT picture (coronar view) of an injured pelvis of a patient before and **b** after the surgery with anatomic reduction. **c** CT picture (coronar view) of an injured pelvis of a patient before and **d** after the surgery without anatomic reduction

Anatomic reduction was achieved in 26 patients (mean age: 46.62; 8 females and 18 males) (see Fig. 3a–b) with the remaining 5 patients (mean age: 53.40; 3 females and 2 males) having a non-anatomic reduction (see Fig. 3c–d). There was unanimous agreement in assessment of reduction between the two experienced pelvic surgeons for all thirty-one cases.

### Evaluation of the subjective post-operational well-being of the patients with and without anatomic reduction

Table 4 shows the overall Majeed score for patients with an anatomic and non-anatomic reduction. Patients with an anatomic reduction had an average Majeed score of 87.15 (± 17.64) compared to an average Majeed score of 72.60 (± 9.29) in the non-anatomic group (*p* < 0.05). A Majeed score showing excellent function was present in 17 patients that had an anatomic reduction compared to 1 patient in the non-anatomic group (*p* < 0.0001).

**Table 4:** Majeed score results

Majeed score [points]	Patients with anatomic reduction (%)	Patients with non-anatomic reduction (%)
> 85 excellent	17 (54.84)	1 (3.23)
70–84 good	4 (12.90)	2 (6.45)
55–69 fair	3 (9.68)	2 (6.45)
< 55 poor	2 (6.45)	–
*n* (total)	26	5

**Fig. 4:**
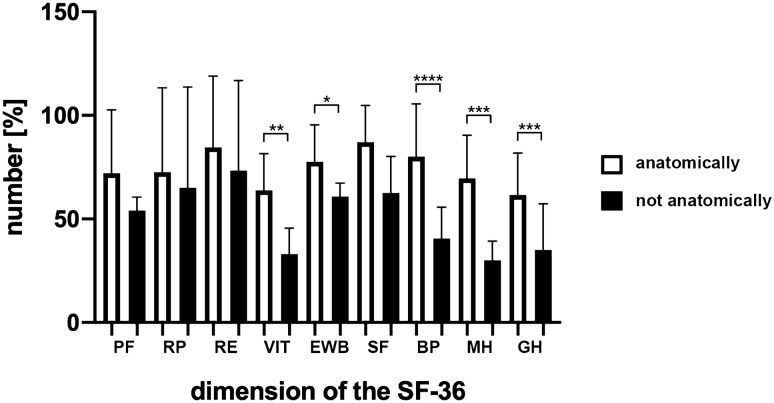
Results of the SF-36- score questionnaire. Shows results of the SF-36- score questionnaire of the patient collective with anatomically correct reduction (white bars) and not anatomically correct reduction (black bars). On the X-axis the different dimensions were applied. *PF *physical functioning, *RP *role limitations due to physical health problems, *RE *role limitations due to personal or emotional problems, *VIT *social functioning, energy/fatigue or vitality, *EWB *emotional well-being, *SF *social functioning, *BP *bodily pain, *MH *general mental health, *GH *general health perceptions. Note the significant differences for VIT (p** = 0.0022), EWB (p* = 0.0135), BP (p**** < 0.0001), MH (p*** = 0.0008) and GH (p** = 0.0158). *n* (anatomically correct = 26); *n* (not anatomically correct = 5)

The separate domains of the SF-36 were compared between patients that had an anatomic and non-anatomic reduction shown in Table 5 below. It is seen that the scores between patients with anatomic and non-anatomic reduction in 6 out of the 9 domains show significant differences between the groups (see Table 5 and Fig. 4).

**Table 5:** SF-36 score results for the populations

SF-36 Domains	Patients with anatomic reduction (%)	Patients with non-anatomic reduction (%)	*p* value
Bodily pain (BP)	90.53	40.50	< 0.0001 (p****)
Mental health (MH)	69.62	30.00	0.0008 (p***)
Social functioning and vitality (VIT)	63.85	33.00	0.0022 (p**)
Emotional well-being (EWB)	77.54	60.80	0.0135 (p*)
General health perception (GH)	61.54	35.00	0.0158 (p***)
Physical function (PF)	72.12	54.00	0.1442 (n. s.)
Social functioning (SF)	89.47	68.75	0.0641 (p**)
Role limitations due to physical health problems (RP)	72.50	65.00	0.8802 (n. s.)
Role limitations due to personal or emotional problems (RE)	87.50	73.34	0.2776 (n. s.)

p* = 0.0135; p** = 0.0022; p** = 0.0641; p*** = 0.0008; p*** = 0.0158; p**** =  < 0.0001; n. s. = 0.1442, 0.8802 and 0.2776

The results of the Numerical rating scale (NRS) revealed a lower indication of pain in the anatomically reduced patient group. The mean NRS value of 1.08 points was found in the group that had an anatomic reduction compared to a mean NRS value of 3 points in the patient group that had a non-anatomic reduction (p*** = 0.0001). The results are summarized in Table 6 below.

**Table 6:** NRS results for the populations

NRS [points] anatomic reduction	1.08 ± 1.44
NRS [points] non-anatomic reduction	3.00 ± 0

### Correlation between Majeed score and SF-36 score

The Pearson product-moment correlation coefficient is a measure of the strength of the linear relationship between two variables. This measure was applied to the sets of data in Tables 4 and 5, indicating that the Majeed score and the SF-36 score are positively correlated in most aspects of the analysis of both, patients with and without anatomic reductions. The Pearson product-moment correlation coefficient values of the patients with anatomic reduction for the nine different aspects outlined above were in the high category PF (*r* = 0.749), MH (*r* = 0.587), EWB (*r* = 0.580), and VIT (*r* = 0.514). A medium correlation was found for RP (*r* = 0.498) and low correlations for BP (*r* = 0.295 and GH *r* = 0.119). No correlation was observed with RE (*r* = 0.085) and SF (*r* = 0.043).

For the patient group without anatomic reduction, strong correlations were found for MH (*r* = 0.773), EWB (*r* = 0.754), BP (*r* = 0.666), GH (*r* = 0.635), RP (*r* = 0.558), VIT (*r* = 0.524) and PF (*r* = 0.504) and a single medium correlation for RE (*r* = 0.353). No correlation was found for SF (*r* = 0.087). These results indicate that the Majeed score and the SF-36 score are positively correlated in the groups with and without anatomically reduced patients.

## Discussion

Pelvic injuries, especially in younger patients are often caused by high-energy trauma [1, 2]. Type B or C injuries with injury of the sacroiliac joint as isolated injuries to the posterior pelvic ring are rare. Percutaneous insertion of sacroiliac screws is a way of stabilizing these injuries. Nevertheless, it is not always possible to achieve a complete anatomic reduction by closed methods. In some cases, the soft tissue does not allow for a greater operational access so that anatomic reduction is not acceptable without harming the patient, i.e. to avoid exposing the patient to an increased risk associated with an open reduction.

It is uncertain whether small radiological incongruences of the sacroiliac joint make a difference in clinical outcome. No data on this is known to date. Subjective quality of life assessment of patients is an increasingly important outcome and can be critically judged using the Short-Form Health Survey (SF-36) [8] and the Majeed Score [7]. The SF-36 is a short, cross-disease measurement tool for assessing the health-related quality of life of patients. It evaluates and allows one to interpret various health aspects including the physical and psychological status of the patient. This tool was used for this present study because its high validity to assess the subjectively felt health status of patients and its worldwide application [9, 10]. Unfortunately, the SF-36 contains no separate reference values for pelvic fractures. Thus, the precasted questionnaire with pelvic-specific questions of the Majeed Score [7] was complemented. It has to be noted that due to the high variability of very frequent injuries which often accompany pelvic fractures [11], studies concerning the value of anatomic reduction to improve the well-being of pelvic fractures have not yet been addressed with patients with only pelvis injuries rather than patients with multiple traumata.

Patients of the two groups, i.e. with or without anatomic reduction, show no significant differences with respect to physical functioning (PF), role limitations due to personal or emotional problems (RE), role limitations due to physical health problems (RP) and social functioning (SF). However, significant differences are clearly evident in the areas of vitality (VIT), bodily pain (BP), emotional well-being (EWB), general health perceptions (GH) as well as general mental health (MH).

To further assess the clinical outcome of the anatomic reduction of the posterior pelvic ring injury, a pelvic-specific scoring system [7] was also applied. This system overlaps with respect to pain assessment with the SF-36 questionnaire, but adds four additional parameters concerning work, sitting, sexuality as well as standing to the list of questions. In accordance with the results of the SF-36 self-assessment of the patients, about 55% of patients with anatomic reductions evaluated their pelvis injury-related health status positively with high scores and only 6.45% were not positive with respect to their health condition. Of the patients without anatomic reductions, only about 3% showed a very good result and 6.45% a fair result. If one compares the examined population with results of other studies, which also used the Majeed score for the evaluation of the outcome the following relationships. Comparable results are shown in the earlier studies [12, 13]. The combined questionnaires used in the present study, i.e. the Majeed scores and the SF-36, show that the data can be positively correlated with respect to nearly all aspects of the presented analysis. This implies that the SF-36 questionnaire, which does not address pelvic-related questions, can indeed be used to assess the quality of life of patients which experienced severe pelvic injuries. Also, the subjective tenderness-related pain sensation at the sacroiliac joint, as quantified by the NRS value, is different (p^***^ = 0.0001) for patients with and without anatomic reduction. In summary, these data allow the conclusion that patients that had an anatomic reduction feel consistently better than patients without.

The present study shows that for patients with sacroiliac disruptions, anatomic reduction seems to have significant beneficial impact on the quality of life of the patients. The importance of maintaining a high quality of life by choosing and evaluating treatment options or measures becomes an increasingly important aspect of patient care. Pelvic injuries represent a rare fracture form, and only few studies concerning pelvic fractures in general have been reported without addressing sacroiliac joint disruptions specifically. These circumstances make it difficult to compare the outcome of the present study with earlier investigations.

Of course, this study also has some limitations. Overall, there are few patients. But the patients in whom the posterior pelvic ring is primarily affected due to a sacroiliac joint injury are not as common. Sometimes dividing into B or C injuries can be difficult. In the present case, there were both B and C injuries. However, no differences concerning the clinical outcome were observed if focused on the posterior pelvic ring. Thus, it was not explicitly referred to B and C injuries. The anterior pelvic ring was also injured in most cases (*n* = 17), be it as a fracture or as an injury to the symphysis.

In this study the focus was on the posterior pelvic ring. Also, due to the small number of patients with pelvic injuries only, the progression healing and the long-term beneficial effects of the applied surgery technique can be evaluated with small patient populations only. Despite the limitation by small patient numbers, this study clearly demonstrates that anatomic reduction after sacroiliac joint injuries is beneficial in maintaining quality of life for patients after surgery.

## Conclusion

Operative treatment of patients with percutaneous reduction of sacroiliac joint disruptions show that anatomic reduction of the posterior pelvic ring has an important and beneficial impact on the subjectively felt well-being of patients. The beneficial impact not only affects bodily pain sensation of patients, but also their general health perceptions such as mental health and emotional well-being, social functioning, vitality as well as physical functioning. Therefore, in cases when anatomic reduction cannot be achieved by closed methods, and small incongruencies persist in the disrupted joint region, an open reduction for better anatomic reconstruction should always be considered. However, if an open reduction is not possible due to a more precarious soft tissue condition, the presence of an unstable post-traumatic condition of the patient or other severe trauma consequences which would interfere with or even prevent an anatomically correct reduction, long-term consequences for the patient cannot be excluded. The results show that patients appear to benefit from anatomical reduction, but since the study has only a relatively small number of cases, additional studies are necessary to further improve the surgical treatment of sacroiliac disruptions with respect to post-surgical well-being of the patients.

## Data Availability

“The datasets supporting the conclusions of this article are included within the article (and its additional files).”

## Abbreviations

BP: Bodily pain
CT: Computer tomography
EWB: Emotional well-being
GH: General health perceptions
ICD: International classification of diseases
MH: General mental health
NRS: Numerical rating scale
N. s.: Not significant
OPS: Operations and procedures key
PF: Physical functioning
RE: Role limitations due to personal or emotional problems
RP: Role limitations due to physical health problems
SF: Social functioning
SF-36: Short-Form Healthy Survey-36 score
VIT: Social functioning, energy/fatigue or vitality
