# Correction to: Anatomic reduction of the sacroiliac joint in unstable pelvic ring injuries and its correlation with functional outcome

**DOI:** 10.1007/s00068-021-01698-w

**Published:** 2021-06-10

**Authors:** Katharina Jäckle, Christopher Spering, Mark-Tilmann Seitz, Sebastian Höller, Marc-Pascal Meier, Franziska Melanie Hahn, Mehool R. Acharya, Wolfgang Lehmann

**Affiliations:** 1 Department for Trauma Surgery, Orthopaedics and Plastic Surgery, University Medical Center Göttingen, Robert-Koch Str. 40, 37075 Göttingen, Germany; 2 Department of Trauma and Orthopaedics, North Bristol NHS Trust, Southmead Hospital, Southmead Rd, Bristol, BS10 5NB UK

## Correction to: European Journal of Trauma and Emergency Surgery 10.1007/s00068-020-01504-z

The article Anatomic reduction of the sacroiliac joint in unstable pelvic ring injuries and its correlation with functional outcome, written by Katharina Jäckle, Christopher Spering, Mark-Tilmann Seitz, Sebastian Höller, Marc-Pascal Meier, Franziska Melanie Hahn, Mehool R. Acharya, Wolfgang Lehmann, was originally published electronically on the publisher’s internet portal on 30. September 2020 without open access. With the author(s)’ decision to opt for Open Choice the copyright of the article changed on 3 May 2021 to © The Author(s) 2020 and the article is forthwith distributed under a Creative Commons Attribution 4.0 International License, which permits use, sharing, adaptation, distribution and reproduction in any medium or format, as long as you give appropriate credit to the original author(s) and the source, provide a link to the Creative Commons licence, and indicate if changes were made. The images or other third party material in this article are included in the article’s Creative Commons licence, unless indicated otherwise in a credit line to the material. If material is not included in the article's Creative Commons licence and your intended use is not permitted by statutory regulation or exceeds the permitted use, you will need to obtain permission directly from the copyright holder. To view a copy of this licence, visit http://creativecommons.org/licenses/by/4.0/.

**Funding** Open access funding enabled and organized by Projekt DEAL.

